# Emergency Hernia Repair Outcomes for Patients With and Without Established Hernia Care With a Surgeon

**DOI:** 10.1001/jamanetworkopen.2025.31290

**Published:** 2025-09-11

**Authors:** Patrick L. Johnson, Cody L. Mullens, Raymond A. Jean, Hugh J. Lindsey, Justin B. Dimick, Mark R. Hemmila

**Affiliations:** 1 Department of Surgery, University of Michigan Medical School, Ann Arbor; 2 Center for Healthcare Outcomes and Policy, University of Michigan, Ann Arbor; 3 Department of Surgery, University of Michigan-Sparrow, Lansing

## Abstract

This cohort study examines the impact of prior established hernia care with a surgeon on postoperative outcomes after emergent hernia repair.

## Introduction

Increased emphasis has been placed on appropriate patient selection and preoperative comorbidity optimization to ensure high-quality hernia repair outcomes in the elective setting.^1^ While optimization efforts are associated with improved complication rates, postponed or deferred hernia repair may lead to incarceration or strangulation, necessitating emergent operation.^2,3^ It is unknown to what extent patients evaluated for elective hernia repair, who did not undergo surgery, contribute to the patient population undergoing emergent repair. Patients with increased comorbidities and hernia complexity may be disproportionately deferred for elective repair and at increased risk for unfavorable outcomes after emergency surgery.

This study evaluated the association of prior established hernia care with a surgeon with postoperative outcomes after emergent hernia repair. We hypothesized that patients with previously established hernia care would have more complications.

## Methods

We performed an observational cohort study of adult patients undergoing emergency hernia repair using data from the Michigan Acute Care Surgery collaborative from January 2022 to December 2024. The database captures patient characteristics, operations performed, clinical outcomes, and hernia-specific information.^4^ This includes multi-institution electronic medical record review to evaluate whether a patient had previously established hernia care with a surgeon (eMethods in Supplement 1). We stratified patients by evidence of prior established hernia care or not. Differences in patient and hernia characteristics were explored with bivariate statistics. We used regression models clustered at the hospital level controlling for patient characteristics and hernia location to assess outcomes. The primary outcome was any complication. Secondary outcomes included mortality, readmission, and mesh use. The University of Michigan institutional review board deemed this study exempt. We followed the Strengthening the Reporting of Observational Studies in Epidemiology (STROBE) reporting guideline. Data were analyzed using Stata/MP version 15 (StataCorp), and statistical significance was set at *P* < .05.

## Results

In this study, 632 patients across 10 hospitals underwent emergency hernia repair, of whom 223 (35%) had prior established hernia care with a surgeon. Patients with established care were more likely to be individuals from minoritized racial and ethnic groups, insured by Medicaid, and smokers (Table 1). Previously evaluated hernias tended to be more complex in size (mean [SD], 6.7 [5.9] cm vs 5.4 [5.4] cm; *P* < .001) and have more history of recurrence (79 [35.4%] vs 92 [22.5%]; *P* < .001) than hernias that were not previously evaluated by a surgeon. Established hernia care was not associated with differences in complications, mortality, or readmission (Table 2). However, established hernia care was associated with lower rates of undergoing mesh repair (39.9% vs 50.9%; adjusted odds ratio, 0.64; 95% CI, 0.47-0.89; *P* = .007).

**Table 1.** Patient Characteristics, by Prior Established Hernia Care With Surgeon

Characteristic	Total	Established hernia care with surgeon		*P* value
		No	Yes	
Patients, No.	632	409	223	
Age, mean (SD)	62.7 (15.7)	64.8 (15.9)	58.9 (14.5)	.002
Sex, No. (%)				
Female	312 (49.4)	220 (53.8)	92 (41.3)	.003
Male	320 (50.6)	189 (46.2)	131 (58.7)	
Race, No. (%)^a^				
Black	113 (17.9)	45 (11.0)	68 (30.5)	<.001
White	481 (76.1)	343 (83.9)	138 (61.9)	
Other	38 (6.0)	21 (5.1)	17 (7.6)	
Hispanic, No. (%)				
Not Hispanic	597 (94.5)	386 (94.4)	211 (94.6)	.90
Hispanic	35 (5.5)	23 (5.6)	12 (5.4)	
BMI, No. (%)				
Normal (≤24.9)	166 (26.2)	112 (27.3)	54 (24.2)	.58
Overweight (25.0-29.9)	149 (23.6)	99 (24.2)	50 (22.4)	
Class I obesity (30.0-34.9)	94 (14.9)	62 (15.2)	32 (14.4)	
Class II obesity (35.0-39.9)	76 (12.0)	48 (11.7)	28 (12.6)	
Class III obesity (≥40.0)	130 (20.6)	80 (19.6)	50 (22.4)	
Unknown	17 (2.7)	8 (2.0)	9 (4.0)	
Insurance, No. (%)				
Commercial	172 (27.2)	113 (27.6)	59 (26.5)	<.001
Medicaid	110 (17.4)	50 (12.2)	60 (26.9)	
Medicare	283 (44.8)	199 (48.7)	84 (37.7)	
Uninsured or other	67 (10.6)	47 (11.5)	20 (9.0)	
ASA score, No. (%)				
1-3	536 (85.1)	350 (85.6)	186 (83.4)	.73
4-5	94 (14.9)	58 (14.2)	36 (16.1)	
Unknown	2 (0.3)	1 (0.2)	1 (0.5)	
No. of comorbidities, No. (%)				
0	109 (17.3)	75 (18.3)	34 (15.3)	.43
1	163 (25.8)	100 (24.5)	63 (28.3)	
2	145 (22.9)	99 (24.2)	46 (20.6)	
3 or more	215 (34.0)	135 (33.0)	80 (35.8)	
Comorbidities, No. (%)				
Prior opioid use	80 (12.7)	45 (11.0)	35 (15.7)	.09
COPD	53 (8.3)	29 (7.1)	24 (10.8)	.11
Smoking	141 (22.3)	70 (17.1)	71 (31.8)	<.001
Diabetes	136 (21.5)	83 (20.3)	53 (23.8)	.31
Hypertension	355 (56.2)	234 (57.2)	121 (54.3)	.47
Functionally dependent health status	29 (4.6)	24 (5.9)	5 (2.2)	.04
History of VTE	69 (10.9)	44 (10.8)	25 (11.2)	.86
Sleep apnea	247 (39.0)	168 (41.1)	79 (35.4)	.16
Hernia characteristics				
Recurrent hernia, No. (%)	171 (27.1)	92 (22.5)	79 (35.4)	<.001
Hernia size (SD), cm^b^	5.8 (5.6)	5.4 (5.4)	6.7 (5.9)	<.001
Hernia location, No. (%)				
Umbilical	130 (20.6)	90 (22.1)	40 (17.9)	<.001
Incisional or ventral	256 (40.5)	158 (38.7)	98 (44.0)	
Inguinal	154 (24.4)	84 (20.6)	70 (30.4)	
Femoral	52 (8.2)	47 (11.5)	5 (2.2)	
Other	39 (6.2)	29 (7.1)	10 (4.5)	

Abbreviations: ASA score, American Society of Anesthesiology Physical Status Classification System; BMI, body mass index; COPD, chronic obstructive pulmonary disease; VTE, venous thromboembolism.

^a^ Race and ethnicity are self-reported by patients, recorded in the electronic medical record (EMR), and extracted by abstractors for this dataset. The race category of other is a collapsed variable that includes Asian, Native Hawaiian or Pacific Islander, Native American or Alaska Native, and Unknown.

^b^ Hernia size calculated as longest dimension between width and length; 306 patients were missing documentation on hernia size in the operative note.

**Table 2.** Perioperative Outcomes and Hernia-Specific Process of Care by Prior Established Hernia Care With a Surgeon

Clinical outcomes	Established hernia care with surgeon, No. (%)		Unadjusted *P* value	Adjusted odds ratio, (95% CI)^a^	*P* value
	No (n = 409)	Yes (n = 223)			
Mesh used	208 (50.9)	89 (39.9)	.008	0.64 (0.47-0.89)	.007
Bowel resection	120 (28.5)	62 (29.6)	.68	1.06 (0.68-1.63)	.80
Mortality^b^	9 (2.2)	5 (2.2)	>.99	1.02 (0.54-1.94)	>.99
Any complication	123 (30.1)	60 (26.9)	.40	0.78 (0.53-1.15)	.27
Readmission	18 (4.4)	10 (4.5)	>.99	1.13 (0.47-2.71)	.84
Unplanned return to ED	40 (9.8)	30 (13.5)	.16	1.25 (0.79-1.98)	.27
Discharge to care facility	69 (16.9)	29 (13.0)	.20	0.78 (0.52-1.17)	.20
Length of stay, mean (SD), h	153.2 (202)	127.1 (153)	.09	NA	NA

^a^ Adjusted values calculated with multivariable regression models clustered at the hospital level using patient sociodemographic data, comorbidity data, and hernia location for covariates.

^b^ Statistical model for mortality used no patient adjustment covariates due to potential for model overfitting.

## Discussion

Our study has 2 principal findings. First, one-third of emergency hernia repairs occur in patients previously evaluated by a surgeon but not repaired. Increased rates of smoking and hernia complexity in this cohort could reflect patterns of delayed hernia care in the elective setting. Identification of established hernia care was limited to documentation available from shared electronic medical record platforms. Thus, the potential for false negatives could suggest that the true prevalence of prior established care is higher than reported.

Second, short-term outcomes were similar for emergent hernia repair in patients with or without prior established care. Mesh use was similarly low to prior work evaluating emergency repair, but significantly lower in patients with established hernia care.^5,6^ High rates of nonmesh repair could indicate increased recurrence risk when previously evaluated hernias are repaired emergently instead of electively. While patient and surgeon decision-making surrounding elective hernia repair is nuanced, our results may be relevant to future optimization algorithms.

We are limited in contextualizing our findings due to inability to characterize why patients did not undergo elective hernia repair after prior evaluation. One patient might have been deferred by the surgeon for tobacco cessation, while another may have declined surgery to pursue watchful waiting. Differences in modifiable characteristics (eg, smoking) between the groups suggest that patient selection by surgeons is a contributing factor. In addition, differences in unmodifiable characteristics (eg, race, insurance status) could indicate potential disparities in elective hernia repair.

## Notes

Supplement 1.
eMethods.

Supplement 2.
Data Sharing Statement
