# Supplementary material for: Emergency Hernia Repair Outcomes for Patients With and Without Established Hernia Care With a Surgeon
Source: JAMA Netw Open. 2025 Sep 11;8(9):e2531290. doi: 10.1001/jamanetworkopen.2025.31290 (PMC12426796; doi:10.1001/jamanetworkopen.2025.31290)
Supplement: Supplement 1. — eMethods. [file jamanetwopen-e2531290-s001.pdf]

Supplemental Online Content

Johnson PL, Mullens CL, Jean RA, Lindsey HJ, Dimick JB, Hemmila MR. Diagnostic testing for celiac disease among patients with abdominal symptoms in primary care: a systematic review. *JAMA Netw Open*. 2025;8(9):e2531290. doi:10.1001/jamanetworkopen.2025.31290

eMethods.

This supplemental material has been provided by the authors to give readers additional information about their work.

© 2025 Johnson PL et al. *JAMA Network Open*.

eMethods.

Data Source and Patient Cohort

Data was obtained from the Michigan Acute Care Surgery (MACS) collaborative. MACS is a Blue Cross Blue Shield of Michigan/Blue Care Network supported collaborative quality initiative capturing emergency general surgery data across ten hospitals. MACS participation is voluntary. MACS activities include data abstraction and collection, benchmark reporting, in-person meetings with unmasking of data, and quality improvement initiatives. Data abstractors are trained using standardized inclusion/exclusion criteria. Data validation visits are conducted annually. We included all patients ≥18 years of age who underwent an abdominal or groin hernia repair. We identified patients undergoing hernia repair by the following International Classification of Diseases, Tenth Revision codes: K40, K41, K42, K43, K45 & K46. An "Unknown" category was used for patients with missing data.

Exposure Variable- Prior Established Hernia Care with a Surgeon

For any patient undergoing abdominal wall or groin hernia repair, data abstractors collect additional hernia specific data. To evaluate for prior established hernia care with a surgeon, data abstractors review all available medical record data at their institution and any institution with electronic medical record sharing agreements. A data abstractor at one facility may have access to all records at that hospital, several other hospitals in the same health system, and numerous hospitals from health systems with electronic medical record sharing agreements. For example, data abstractors at the senior author's hospital have access to data from the three largest health systems in the state.

© 2025 Johnson PL et al. *JAMA Network Open*.
