# Supplementary material for: Emergency Hernia Repair Outcomes for Patients With and Without Established Hernia Care With a Surgeon
Source: JAMA Netw Open. 2025 Sep 11;8(9):e2531290. doi: 10.1001/jamanetworkopen.2025.31290 (PMC12426796; doi:10.1001/jamanetworkopen.2025.31290)
Supplement: Supplement 2. — Data Sharing Statement [file jamanetwopen-e2531290-s002.pdf]

Data Sharing Statement

Johnson. Emergency Hernia Repair Outcomes for Patients With and Without Established Hernia Care With a Surgeon. *JAMA Netw Open*. Published September 11, 2025. doi:10.1001/jamanetworkopen.2025.31290

Data
Data available: No

Additional Information
Explanation for why data not available: A data dictionary defining each field in the data set can be made available. However, the data set belongs to the individual participant hospitals and the MACS collaborative. It is not a publicly available data set.
